# Identifying Active Compounds and Targets of *Fritillariae thunbergii* against Influenza-Associated Inflammation by Network Pharmacology Analysis and Molecular Docking

**DOI:** 10.3390/molecules25173853

**Published:** 2020-08-25

**Authors:** Minjee Kim, Ki Hoon Park, Young Bong Kim

**Affiliations:** Department of Biomedical Science and Engineering, Konkuk University, Seoul 05029, Korea; mj0411@konkuk.ac.kr (M.K.); mhjhyun@konkuk.ac.kr (K.H.P.)

**Keywords:** *Fritillariae thunbergii*, influenza, inflammation, network pharmacology, molecular docking, systems biology

## Abstract

Complications due to influenza are often associated with inflammation with excessive release of cytokines. The bulbs of *Fritillariae thunbergii* (FT) have been traditionally used to control airway inflammatory diseases, such as bronchitis and pneumonia. To elucidate active compounds, the targets, and underlying mechanisms of FT for the treatment of influenza-induced inflammation, systems biology was employed. Active compounds of FT were identified through the TCMSP database according to oral bioavailability (OB) and drug-likeness (DL) criteria. Other pharmacokinetic parameters, Caco-2 permeability (Caco-2), and drug half-life (HL) were also identified. Biological targets of FT were retrieved from DrugBank and STITCH databases, and target genes associated with influenza, lung, and spleen inflammation were collected from DisGeNET and NCBI databases. Compound-disease-target (C-D-T) networks were constructed and merged using Cytoscape. Target genes retrieved from the C-D-T network were further analyzed with GO enrichment and KEGG pathway analysis. In our network, GO and KEGG results yielded two compounds (beta-sitosterol (BS) and pelargonidin (PG)), targets (PTGS1 (COX-1) and PTGS2 (COX-2)), and pathways (nitric oxide, TNF) were involved in the inhibitory effects of FT on influenza-associated inflammation. We retrieved the binding affinity of each ligand-target, and found that PG and COX-1 showed the strongest binding affinity among four binding results using a molecular docking method. We identified the potential compounds and targets of FT against influenza and suggest that FT is an immunomodulatory therapy for influenza-associated inflammation.

## 1. Introduction

Seasonal influenza A virus (IAV) is an infectious RNA virus that causes acute respiratory infection, leading to high morbidity and mortality during pandemics [1]. Complications or deaths due to IAV infections are often associated with inflammation events, such as the aggressive pro-inflammatory response, and increased cytokine production [2]. IAV primarily targets the respiratory tract, resulting in epithelial damage, pulmonary infiltration, and hypoxemia, leading to acute respiratory distress syndrome (ARDS) [1]. In the event of mild infection, the inflammatory response is crucial for the clearance of virus from the lung, and the host has limited resistance to the virus to restore homeostasis [3]. However, in serious infections, immune systems of hosts become hyperactive with excessive release of cytokines, described as a cytokine storm [3,4]. Moreover, several inflammatory cytokines and chemokines are secreted into the circulation, potentially resulting in multi-organ dysfunction [3]. Upon viral infection, lung antigen-presenting cells (APCs) acquire viral antigens and undergo a maturation process that induces migration to local draining lymph nodes [5]. IAV-specific effector CD8 T cells are found in lymphoid sites, such as the spleen, which was reported to be a contributor to the immune response to respiratory infection [5]. Herein, we constructed a network of influenza-, lung-inflammation-, and spleen-inflammation-associated targets to elucidate targets of influenza-induced inflammation.

Bulbs from *Fritillariae thunbergii* (FT) are traditionally used to treat airway inflammatory diseases, such as bronchitis and pneumonia [6]. In a recent study, we demonstrated antiviral effects of FT extracts against influenza [7]. However, the specific components exerting these effects and molecular mechanisms remain unclear. Here, we examined the efficacy of FT in the treatment of influenza-associated inflammations and further identified its active compounds and targets.

Due to complex matrices of plant extracts, elucidation of the underlying molecular mechanisms is difficult because of the synergistic effects of the active compounds and multiple therapeutic targets involved. Network pharmacology is an emerging approach that emphasizes the concept of “network target, multicomponent therapeutics” [8]. The technique uses compound-disease-target network visualization to evaluate multi-target mechanisms of plant extracts [8,9,10,11]. Computational research is another promising time-saving alternative to experimental research that may aid in identification of novel compounds and targets [12,13]. 

In an attempt to understand the molecular mechanism of action of FT against influenza-associated inflammation, three compound-disease-target (C-D-T) networks (influenza, lung inflammation, and spleen inflammation) were constructed and merged into a single network to yield an influenza-associated inflammation network system. We also observed the binding affinity of each ligand-target using a molecular docking method to identify the potential compounds and targets of FT.

## 2. Results

### 2.1. Active Compounds Selection Using Physicochemical Characteristics

Overall, 17 compounds were screened using the TCMSP database. Compounds that satisfied oral bioavailability (OB) ≥ 30% and drug-likeness (DL) ≥ 0.18 criteria suggested by the TCMSP database were considered potent [14]. Using the criteria, 7 out of the original 17 compounds were retrieved: pelargonidin (PG), beta-sitosterol (BS), peimisine (PM), zhebeiresinol (ZBR), ziebeimine (ZBM), 6-methoxyl-2-acetyl-3-methyl-1,4-naphthoquinone-8-O-beta-D-glucopyranoside (6GP), and chaksine (CS) (Table 1). Based on seven compounds, Caco-2 permeability (Caco-2) and drug half-life (HL) data were collected to determine pharmacokinetics of FT compounds. Our results indicated that BS showed the highest permeability and drug half-life.

### 2.2. Compounds Associated with Biological Targets

Seven compounds identified from the TCMSP database were further analyzed to identify biological target genes. In total, 44 targets were linked to five of the identified compounds (PG, BS, PM, ZBR and 6GP) from the DrugBank database. Nine target genes were identified with one compound (BS) with a combined score >700 from the STITCH database. Between the two databases, there were six overlapping targets, and 47 targets associated with the five compounds were finally retrieved (Table 2).

### 2.3. Compound-Disease-Target (C-D-T) Networks

**Figure 1:**
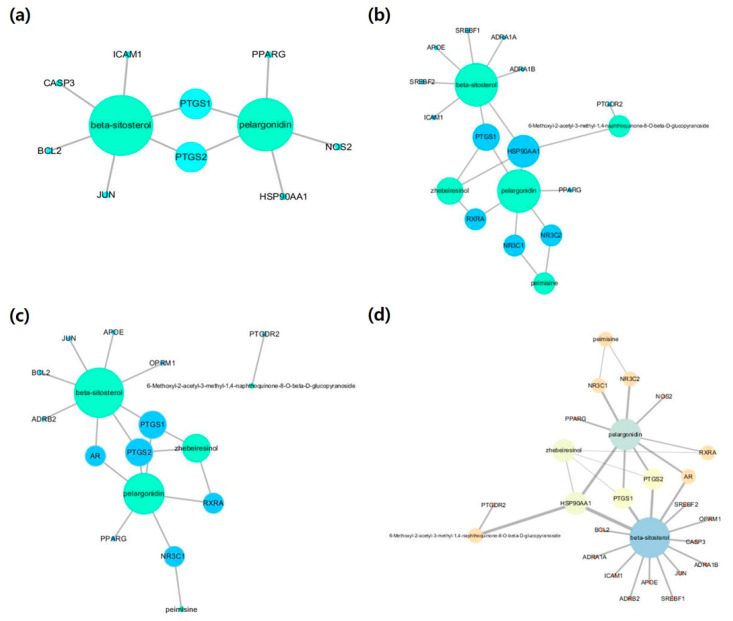
Compound-disease-target networks: (**a**) compound-influenza-target, (**b**) compound-lung-inflammation-target, (**c**) compound-spleen inflammation-target and (**d**) compound-merged targets. The size of the node indicates the degree.

Influenza-, lung-inflammation-, and spleen-inflammation-associated target genes were retrieved from Genecards, DisGeNET, and NCBI databases. From the 1317 influenza target genes retrieved, compound-associated targets were selected. The size of nodes were proportional to degree centrality. According to the compound-influenza-target (C-D-T1) network (Figure 1a), two compounds (BS and PG) and nine target genes (BCL2, CASP3, HSP90AA1, ICAM1, JUN, NOS2, PPARG, PTGS1, and PTGS2) were obtained with 11 nodes and 11 edges. A number of lung-inflammation- or pneumonia-associated genes were searched and identified from the databases. Our compound-lung inflammation-target (C-D-T2) network (Figure 1b) revealed five compounds (PM, PG, BS, 6GP, and ZBR) associated with 12 targets (NR3C1, AR, ADRB2, APOE, BCL2, PPARG, PTGDR2, PTGS1, PTGS2, JUN, OPRM1, and RXRA), with 17 nodes and 19 edges. For spleen inflammation, a total of 1213 target genes were retrieved from the databases. From the third network, a compound-spleen inflammation-target (C-D-T3) network (Figure 1c) with 19 nodes and 21 edges, five compounds (PM, PG, BS, 6GP, and ZBR) and 13 targets (ADRA1A, ADRA1B, APOE, HSP90AA1, ICAM1, NR3C1, NR3C2, PPARG, PTGDR2, PTGS1, RXRA, SREBF1, and SREBF2) were identified. All three networks were merged to identify influenza-, lung-inflammation-, and spleen-inflammation-associated targets (Figure 1d). From our merged network with 26 nodes and 32 edges, five compounds (BS, PG, ZBR, PM, and 6GP) and 21 targets (HSP90AA1, PTGS1, PTGS2, NR3C2, NR3C2, AR, RXRA, ADRA1A, ADRA1B, SREBF1, SFREBF2, ADRB2, APOE, PTGDR2, OPRM1, BCL2, CASP3, ICAM1, JUN, NOS2, and PPARG) were retrieved. Two compounds (BS and PG) and targets (prostaglandin-endoperoxide synthase 1 (PTGS1) and prostaglandin-endoperoxide synthase 2 (PTGS2)) showed the highest degree centrality.

### 2.4. GO Enrichment and KEGG Pathway Analysis

**Figure 2:**
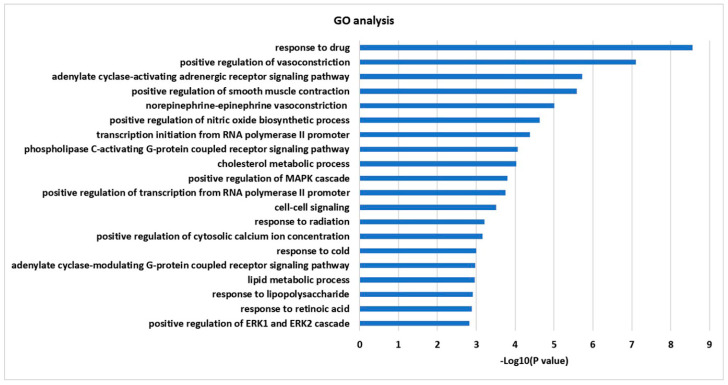
GO enrichment analysis for 21 targets.

Twenty-one targets retrieved from network were further analyzed using DAVID bioinformatics resources for GO and KEGG enrichment analysis. The top 20 enriched terms (*p* < 0.05) were obtained from identified targets in the GO biological process. The order of importance was ranked based on the Log10 (*p*-value) with a bar chart. Specifically, these targets were enriched with inflammation, positive regulation of the nitric oxide biosynthetic process, positive regulation of the MAPK cascade, response to lipopolysaccharide (LPS), and response to retinoic acid (Figure 2). 

**Figure 3:**
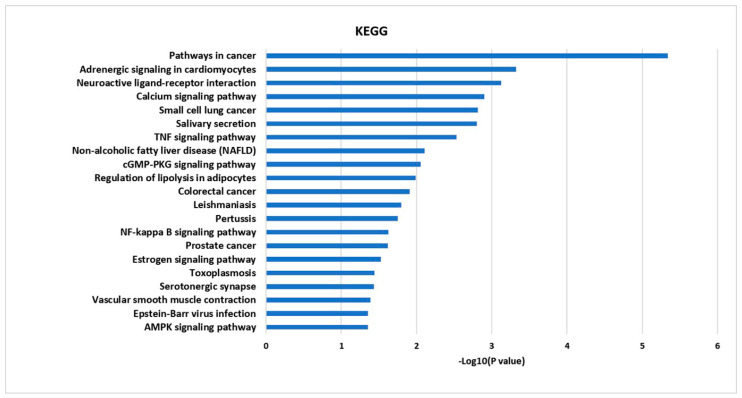
The KEGG pathway enrichment analysis for 21 targets.

To investigate integral regulation of influenza-associated inflammation by FT, top 20 KEGG pathways were obtained from 21 targets, including NF-κB and TNF signaling, which are associated with inflammation, small-cell lung cancer and salivary secretion in relation to respiratory tract disease and function (Figure 3).

### 2.5. Molecular Docking Analysis

A ligand-target docking approach was used to analyze structural complexes of targets (PTGS1, also known as COX-1: PDB no. 6Y3C, and PTGS2, also known as COX-2 PDB no. 5F1A) with ligands (BS and PG) using Autodock Vina system in PyRx virtual screening tool. We retrieved the binding affinity of each ligand-target and found that PG and COX-1 showed the strongest binding affinity among four binding results (Table 3).

**Figure 4:**
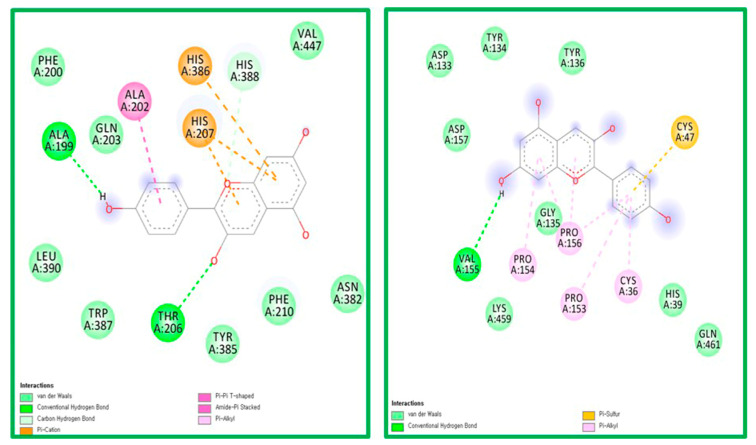
The ligand-receptor interaction screening of PG-COX-1 and PG-COX-2.

**Figure 5:**
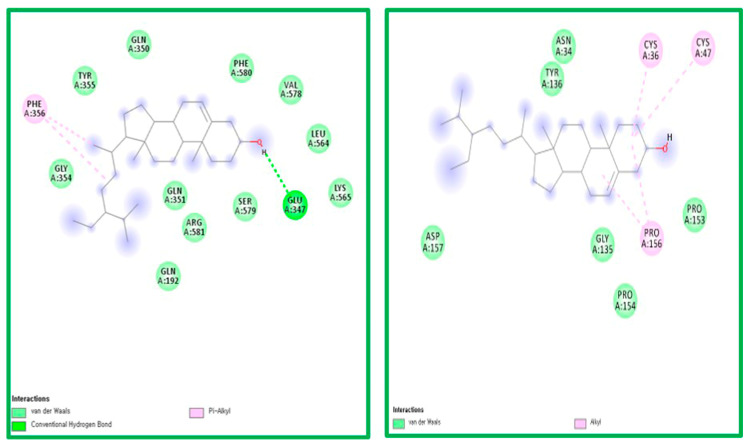
The ligand-receptor interaction screening of BS- COX-1 and BS-COX-2.

The ligand-receptor interaction was screened using Discovery Studio Visualizer (Figure 4 and Figure 5). Compound PG was found to interact with COX-1 amino acids HIS207 via π-cation, HIS388 via carbon-hydrogen bond, ALA202 via π-alkyl, and ALA199 via hydrogen bond. PG was also found to interact with COX-2 amino acids CYS47 via π-sulfur, PRO156, PRO153, PRO154, and CYS36 via π-alkyl, and generated hydrogen bonding with VAL155 (Figure 4).

Compound BS was found to interact with COX-1 amino acid PHE356 via π-alkyl and GLU347 via hydrogen bonding. PG was also found to interact with COX-2 amino acids CYS47, CYS 36, and PRO156 via alkyl bonds (Figure 5).

## 3. Discussion

Influenza-induced inflammation contributes to disease severity, leading to high morbidity and mortality rates [1,4]. IAV targets the epithelial cells of the upper and lower respiratory tract, and the first responders to viral infection are tissue-resident alveolar macrophages in the lung [1]. The virus induces high levels of pro-inflammatory cytokines and chemokines, producing massive infiltration and damage in lung tissues [1]. Pro-inflammatory cytokines, such as interferons, interleukins, and tumor necrosis factor (TNF), are the main regulators of the lung environment during infection [3]. Release of pro-inflammatory cytokines induces differentiation into monocyte-derived alveolar macrophages and dendritic cells (DCs), including TNF/inducible nitric oxide synthase (iNOS)-producing DCs that promote IAV-related alveolar injury [15]. After pulmonary influenza infection, late-activator antigen-presenting cells (LAPC) enter the lung and acquire viral antigens that migrate to the spleen and draining lymph nodes [5,16]. 

In this study, we conducted network pharmacology, systems biology, and molecular docking to acquire system-level information on FT activity against influenza-associated inflammation. FT has been traditionally used to treat airway diseases [17]. Previously, we observed antiviral effects of FT against influenza [7], and the current study was conducted to further identify novel active compounds and therapeutic targets of FT. From the TCMSP database, seven potential compounds were screened according to the suggested pharmacokinetic factors. DrugBank and STITCH databases were applied to retrieve compound-associated biological targets, leading to the identification of 47 final targets. A holistic C-D-T network was generated by merging three networks to determine the factors involved in influenza-associated inflammation, which yielded two main active compounds (BS and PG) and main targets (PTGS1 and PTGS2). Based on the collective networks, GO and KEGG results, PTGS2 (prostaglandin-endoperoxide synthase 2, also known as COX-2), nitric oxide (iNOS). and tumor necrosis factor α (TNFα) were identified as being involved in influenza-induced inflammation. 

Anti-inflammation is one of the key pharmacological effects of FT [18]. In our data search, a total of seven compounds were identified (PG, BS, PM, ZBR, ZBM, 6GP, and CS). FT compounds other than in our study, such as isoverticine, puqiedine, N-demethylpuqietinone, and 2-monopalmitin, showed anti-inflammatory effects by reducing NF-κB expression in the human kidney cells (HEK293) [19], and peimine was shown to exert anti-inflammatory effects by blocking T lymphocytes-induced immune responses [20]. From our seven compounds, ZBR was also reported to reduce NF-κB expression in HEK293 cells [19]. BS and PG were the final two compounds identified from our network pharmacology study. BS, one of the most abundant plant phytosterols, is stigmast-5-ene-substituted by a beta-hydroxy group at position three and derives from a hydride of a stigmastane [21]. BS was reported to suppress the chemotactic cytokine genes in cystic fibrosis bronchial epithelial cells [22] and inhibit epithelial-to-mesenchymal transition in lung alveolar cells, consistent with our GO and KEGG results [23]. Recent pathophysiology-associated studies showed that BS attenuates rheumatoid inflammation in mice via modulating macrophages [24], and reduced expression of inflammatory cytokines and TNFα in brain inflammation-induced Wistar rats [25]. PG is an anthocyanidin that is flavylium-substituted by hydroxy groups at positions three, five, seven and four, which is actually the sole chemical species in fairly acidic aqueous solution [26]. Recently, PG showed to exert anti-inflammatory effects by suppressing the production of TNFα in LPS-induced vascular inflammation in vein endothelial cells, in accordance with our pathway results [27]. To the best of our knowledge, this is the first study to identify two compounds (BS and PG) that could be potent for influenza-associated inflammation.

TNFα is one of the most extensively characterized cytokines involved in multiple effects, such as the activation of inflammatory responses, stimulation of adaptive immunity, and apoptosis [28]. The TNFα signaling pathway was identified from our KEGG pathway results and further confirmed in the animal model experiments. In the case of IAV-induced inflammation, TNFα blocking agents were reported to reduce lung inflammation and morbidity after challenge [29]. COX-2 is an enzyme that converts arachidonic acid in prostaglandins that may contribute to inflammation [28]. Increased COX-2 expression was reported in pandemic H5N1 influenza virus in vitro and in lung tissue samples obtained from patients who died of H5N1 disease [30]. Another study showed that inhibition of COX-2 by paracetamol or a selective inhibitor (celecoxib) during H1N1 and H3N2 infection prevented lung immunopathology without affecting virus clearance in mice [31]. iNOS, a key molecule in combating viral infection, has been reported to mediate apoptosis and promote inflammation. One study reported high levels of mortality and associated pathology with increased iNOS expression in influenza-infected chickens [32]. Therefore, FT-induced reduction of TNFα, COX-2, and iNOS may exert protective effects against influenza-induced inflammatory responses. 

In principle, we designed an integrative approach to identify potential compounds and targets of FT associated with influenza-induced inflammation. Our results support the development of FT as an immunomodulatory therapy aimed at suppressing the unwanted inflammatory response triggered by IAV infection. The theory is that administration of such anti-inflammatory drugs with anti-viral effects during severe cases of flu may reduce patient symptoms and favorably modify the prognosis of the infection. In this respect, therapeutic application of FT could lead to a decrease in the number of hospitalizations and complications associated with IAV infection.

### 3.1. Prediction of Potential Active Compounds of FT

Compounds of FT were searched using the Traditional Chinese Medicine Systems Pharmacology database (TCMSP) (http://tcmspw.com/tcmsp.php). Active compounds were selected using the in silico physicochemical model within the database according to oral bioavailability (OB) and drug-likeness (DL), Caco-2 permeability (Caco-2) and drug half-life (HL) results suggested by the TCMSP database (ver.2.3). 

### 3.2. Potential Therapeutic Targets Analysis

Target genes associated with the potential compounds were further investigated using the DrugBank (https://www.drugbank.ca, version 5.1.4) and STITCH (http://stitch.embl.de/, version 5.0) databases. All searches were performed with the *Homo sapiens* species setting. Influenza-, lung-inflammation-, and spleen-inflammation-associated target genes (*H. sapiens*) were obtained from the DisGeNET (https://www.disgenet.org/, version 7.0) and NCBI databases (https://www.ncbi.nlm.nih.gov/gene). Three compound-disease-target (C-D-T) networks (influenza, lung inflammation, and spleen inflammation) were constructed using Cytoscape. All three C-D-T networks were merged into one to identify potential influenza-inflammation associations. 

### 3.3. Pathway Analysis and Network Construction 

Information on pathways highly associated with target proteins was retrieved from Database for Annotation, Visualization and Integrated Discovery (DAVID version 6.8) and the Kyoto Encyclopedia of Genes and Genomes (KEGG) database. 

### 3.4. Molecular Docking Analysis

A ligand-target docking approach was used to analyze structural complexes of the target with ligands to understand target specificity. The three-dimensional structure of target was downloaded from the RCSB protein data bank. The chemical structures of ligands were obtained from PubChem database and converted to PDB file using Avogadro program (version 1.90.0). Docking was carried out by AutoDock Vina (version 1.1.2) option in Pyrx (version 0.9.6) tool based on scoring functions. Computer Atlas of Surface Topology of protein (CASTp) server (version 3.0) was used to locate active site of targets. 

## Figures and Tables

**Table 1:** Active compounds screening.

	Active Compounds						
	PG	BS	PM	ZBR	ZBM	6GP	CS
MW	271.26	414.79	427.69	280.3	413.71	422.42	450.66
OB	37.99	36.91	57.4	58.72	64.25	33.31	65.63
DL	0.21	0.75	0.81	0.19	0.7	0.57	0.66
Caco-2	0.31	1.32	0.18	0.53	0.81	−1.21	−0.01
HL	0.48	5.37	14.39	3.32	7.81	31.01	0.58

MW: molecular weight, OB: oral bioavailability, DL: drug-likeness, Caco-2 permeability (Caco-2), Drug half-life (HL), PG: Pelargonidin, BS: beta-sitosterol, Peimisine: PM, Zhebeiresinol: ZBR, Ziebeimine: ZBM, 6-methoxyl-2-acetyl-3-methyl-1,4-naphthoquinone-8-O-beta-D-glucopyranoside: 6GP, Chaksine: CS.

**Table 2:** Active compounds and associated targets.

Compound	Target
BS	ABCB11, ABCG5, ABCG8, ADRA1A, ADRA1B, ADRB2, APOE, BCL2, CASP3, CHRM1, CHRM2, CHRM3, CHRM4, CHRNA2, CYP7A1, DRD1, GABRA1, HSP90AA1, HTR2A, ICAM1, JUN, KCNH2, MAP2, NCOA2, OPRM1, PDE3A, PGR, PON1, PTGS1, PTGS2, SCN5A, SLC6A4, SREBF1, SREBF2
PG	ACHE, AR, CA2, HSP90AA1, NCOA1, NCOA2, NOS2, NR3C1, NR3C2, PGR, PPARG, PTGS1, PTGS2, RXRA
ZBR	ADRB2, GABRA1, HSP90AA1, PDE3A, PTGS1, PTGS2, RXRA, SCN5A
6GP	CA2, ESR1, F7, HSP90AA1, NCOA2, PTGDR2, TOP2B
PM	NR3C1, NR3C2

**Table 3:** Ligand-target binding affinity.

Ligand-Target	Binding Affinity (kcal/mol)
PG-COX-1	−8.6
PG-COX-2	−7.9
BS-COX-1	−6.9
BS-COX-2	−7.4
